# Ratio of carbon dioxide veno-arterial difference to oxygen arterial-venous difference is not associated with lactate decrease after fluid bolus in critically ill patients with hyperlactatemia: results from a prospective observational study

**DOI:** 10.1186/s12871-023-01993-6

**Published:** 2023-01-31

**Authors:** Keitiane Kaefer, Charalampos Pierrakos, Thomas Nguyen, Dimitrios Velissaris, Rachid Attou, Jacques Devriendt, Sabino Scolletta, Fabio Silvio Taccone

**Affiliations:** 1 Intensive Care Department, Brugmann University Hospital, Université Libre de Bruxelles, Brussels, Belgium; 2 Internal Medicine Department, University Hospital of Patras, Patras, Greece; 3 Department of Emergency-Urgency and Organ Transplantation, Anesthesia and Intensive Care, University Hospital of Siena, Siena, Italy; 4 Intensive Care Department, Erasme Hospital, Université Libre de Bruxelles, Brussels, Belgium

**Keywords:** Fluid challenge, Fluid expansion, Oxygen consumption, Tissue hypoxia, PCO_2_ gap, Veno-arterial carbon dioxide gap, Venous oxygen saturation, Tissue perfusion, Lactate clearance

## Abstract

**Background:**

High ratio of the carbon dioxide veno-arterial difference to the oxygen arterial-venous difference (P_va_CO_2_/C_av_O_2_) is associated with fluid bolus (FB) induced increase in oxygen consumption (VO_2_). This study investigated whether P_va_CO_2_/C_av_O_2_ was associated with decreases in blood-lactate levels FB in critically ill patients with hyperlactatemia.

**Methods:**

This prospective observational study examined adult patients in the intensive care unit (ICU) with lactate levels > 1.5 mmol/L who received FBs. Blood-lactate levels were measured before and after FB under unchanged metabolic, respiratory, and hemodynamic conditions. The primary outcome was blood-lactate levels after FB. Significant decreases in blood-lactate levels were considered as blood-lactate levels < 1.5 mmol/L or a decrease of more than 10% compared to baseline.

**Results:**

The study enrolled 40 critically ill patients, and their median concentration of blood lactate was 2.6 [IQR:1.9 − 3.8] mmol/L. There were 27 (68%) patients with P_va_CO_2_/C_av_O_2_ ≥ 1.4 mmHg/ml, and 10 of them had an increase in oxygen consumption (dVO_2_) ≥ 15% after FB, while 13 (32%) patients had P_va_CO_2_/C_av_O_2_ < 1.4 mmHg/ml before FB, and none of them had dVO_2_ ≥ 15% after FB. FB increased the cardiac index in patients with high and low preinfusion P_va_CO_2_/C_av_O_2_ (13.4% [IQR: 8.3 − 20.2] vs. 8.8% [IQR: 2.9 − 17.4], *p* = 0.34). Baseline P_va_CO_2_/C_av_O_2_ was not found to be associated with a decrease in blood lactate after FB (OR: 0.88 [95% CI: 0.39 − 1.98], *p* = 0.76). A positive correlation was observed between changes in blood lactate and baseline P_va_CO_2_/C_av_O_2_ (*r* = 0.35, *p* = 0.02).

**Conclusions:**

In critically ill patients with hyperlactatemia, P_va_CO_2_/C_av_O_2_ before FB cannot be used to predict decreases in blood-lactate levels after FB. Increased P_va_CO_2_/C_av_O_2_ is associated with less decrease in blood-lactate levels.

**Supplementary Information:**

The online version contains supplementary material available at 10.1186/s12871-023-01993-6.

## Introduction

Blood-lactate concentrations are frequently measured at the bedside in critically ill patients, and high lactate levels have been widely used as a marker of tissue hypoxia [1]. However, blood lactate is not frequently used as a trigger to administrate fluid bolus (FB) in this setting [2] unless very high lactate concentrations are observed. Circulating lactate levels are the product of the balance between lactate generation, metabolism, and clearance rate. As such, hyperlactatemia may not reflect only tissue hypoxia, but also the equilibration between production and utilization of lactate [3]. Therefore, giving FB based on only high lactate levels might lead to an inadequate or excessive fluid administration in critically ill patients [4, 5].

The ratio of the veno-arterial difference of carbon dioxide partial pressure to over the artero-venous difference in oxygen content (P_va_CO_2_/C_av_O_2_) has been suggested as a marker of tissue hypoxia that can be easily measured at bedside [6]. An increased value represents an inequivalence between global CO_2_ production and oxygen consumption, which is typically found in anaerobic metabolism [6, 7]. Thus, FB administration in patients with high P_va_CO_2_/C_av_O_2_ may have significant metabolic effects as the expected improvement in oxygen delivery can decrease anaerobic metabolism and lactate production.

Previous studies have demonstrated that high P_va_CO_2_/C_av_O_2_ was associated with an increase in oxygen consumption (VO_2_) after FB [8, 9]. However, the association of baseline P_va_CO_2_/C_av_O_2_ in significant lactate decreases after FB has not been established yet and its role in treating patients with hyperlactatemia is under investigation [10]. The goal of this study was to test the hypothesis that a high P_va_CO_2_/C_av_O_2_ is associated with decreasing blood-lactate levels during FB and whether it has clinical utility to guide fluid treatment in this regard. To do so, we evaluated blood-lactate kinetics and preinfusion P_va_CO_2_/C_av_O_2_ in critically ill patients with hyperlactatemia who received FB.

## Methods

### Design and setting

This is prospective observational study enrolled patients treated in the 33-bed Intensive Care Unit (ICU) of Brugmann Hospital in Brussels, Belgium. The patients received FB between January and June 2015. Approval was obtained from the local Ethics Committee (CE2014/122), and written informed consent was obtained by the patient’s next of kin accordingly. The decisions about the indication, type, amount, and speed of FB were made by the treating physician.

### Inclusion and exclusion criteria

Critically ill adult patients (> 18 years of age) with blood-lactate levels > 1.5 mmol/L who received FBs in less than 50 min were considered eligible for the study [11]. The exclusion criteria were: 1) a lack of jugular or subclavian venous catheter and arterial catheter; 2) FB using Ringer’s lactate solutions, to avoid any increasing of blood lactate due to fluid administration; 3) other interventions within 30 min before FB or during FB (i.e., introduction or increase of inotrope dosage, ventilator mode changes, or the initiation of mechanical ventilation); 4) patients on extracorporeal membrane oxygenation (ECMO) support; 5) patients with diabetic ketoacidosis (blood glucose > 300 mg/dL, positive urine ketone test, pH < 7.35 in arterial blood gas analysis); 6) treatment with metformin within 48 h before FB; 7) clinical suspicion of epileptic crisis or physical effort or as a cause of hyperlactatemia; and 8) patients with diagnosis of metastatic malignancy or leukaemia or malaria.

### Data and sample collections

Demographics, the type of fluids used for FB, concomitant treatments (mechanical ventilation, inotropic agents), and laboratory data were collected for each patient. The Acute Physiology and Chronic Health Evaluation (APACHE) II score upon admission was used to assess disease severity. Before and after FB, we measured the cardiac index (CI) using Doppler echocardiography and performed arterial and central venous blood gas analyses (which were sampled simultaneously), including haemoglobin, arterial and venous oxygen pressure (P_a_O_2_ and P_v_O_2_, respectively), and oxygen saturation (S_a_O_2_ and S_cv_O_2_).

The arterial and venous oxygen content (C_a_O_2_, C_v_O_2_), oxygen delivery (DO_2_), oxygen consumption (VO_2_), and the oxygen extraction ratio (OER) were computed using validated formulas [12]. Also, the venous-to-arterial carbon dioxide tension (P_va_CO_2_) and the venous-to-arterial carbon dioxide tension/arterial-venous oxygen content difference ratio (P_va_CO_2_/C_av_O_2_) were calculated. Each patient was assessed once.

### Definitions and outcome

Sepsis and septic shock were defined according to Sepsis-3 definition [13]. Enhanced oxygen extraction was defined as OER > 40% and/or S_cv_O_2_ < 60%. A significant decrease in lactate concentrations was defined as: a) post-FB lactate < 1.5 mmol/L) or b) a decrease of more than 10% from baseline values [14]. High P_va_CO_2_/C_av_O_2_ was defined as a value ≥ 1.4 [15]. A significant increase in VO_2_ was defined as an augmentation (dVO_2_) ≥ 15% from baseline [8]. The primary outcome was the predictive value of P_va_CO_2_/C_av_O_2_ for significant lactate decrease after FB. Secondary outcomes included: a) the association of lactate and P_va_CO_2_/C_av_O_2_ before and after FB and b) the association of changes in VO_2_ and blood lactate changes after FB.

### Statistical analysis

Statistical analyses were done in R through the R-studio interface (www.r-project.org, R version 3.3.1). Descriptive statistics were computed for all study variables. A Kolmogorov–Smirnov test was used, and histograms and normal-quantile plots were examined to verify the normality of the distribution of continuous variables. Absolute changes (Δ = After FB value – Before FB value) and relative changes (d = [(After FB value – Before FB value)/ Before FB value] × 100) of different variables were evaluated. Discrete variables were expressed as counts (percentages) and continuous variables as the means ± SDs or medians with interquartile ranges (IQR).

A student’s t-test or Wilcoxon signed-rank test was performed as appropriate. Categorical variables were compared using Fisher’s exact test. Univariate logistic regression analysis was done to assess the association of P_va_CO_2_/C_av_O_2_ with a significant decrease in blood-lactate levels after FB. Sensitivity analysis was also performed for a significant increase in dVO_2_ after FB. Odds ratios (OR) with 95% confidence intervals (CI) were computed. Spearman’s correlation and scatter diagrams were used to assess correlations between values. Statistical significance was defined using *p* < 0.05.

## Results

### Study population

Out of a total of 80 patients who received FBs during the study period, 40 patients (age 71 ± 15 years) met the entry criteria and were included in the analysis (Figure S1). There were 25 patients (58%) who were admitted for medical reasons, and the majority of the patients (*n* = 24, 55%) had sepsis at the time of FB. Colloids were used in 22 patients (54%) (Geloplasma®, Fresenius-Kabi AG, Bad Homburg, Germany), and crystalloids were used in 18 (46%) (Plasma-Lyte A, Baxter Healthcare, Deerfield, IL). The elapsed time between the baseline sample and the sample after the FB was 33 (27 − 42) min; the main reason for FB was persistent elevated levels of blood lactate (Table S1).

Before FB, high P_va_CO_2_/C_av_O_2_ ≥ 1.4 was observed in 27 patients (68%). No significant differences were found between patients with high and low P_va_CO_2_/C_av_O_2_ at baseline (Table 1) except for a higher incidence of diabetes and pulmonary infection in patients with low P_va_CO_2_/C_av_O_2_. No significant differences in changes in systemic haemodynamics were observed between the two groups (Table 2). FB increased central venous pressure and cardiac output in both group of patients.

**Table 1:** Characteristics of the study population according to baseline ratio of veno-arterial carbon dioxide difference to arterio-venous oxygen difference (P_va_CO_2_/C_av_O_2_): “low group” has P_va_CO_2_/C_av_O_2_ < 1.4 mmHg/mL, and “high group” has P_va_CO_2_/C_av_O_2_ ≥ 1.4 mmHg/mL. Data are expressed as the median (25–75% percentiles) or count (%)

	Low Group	High Group	*p* values
No of patients	13	27	
Demographic characteristics			
Age, years	66 (55 − 80)	71 (62 − 84)	0.29
APACHE II Score	24 (23 − 27)	24 (17 − 32)	0.98
Comorbidities			
Diabetes, n (%)	7 (53)	4 (26)	0.02
Cirrhosis, n (%)	2 (15)	5 (18)	0.99
Sepsis, n (%)	11 (84)	14 (51)	
Pulmonary infection	9 (81)	9 (33)	0.04
Shock, n (%)	3 (23)	10 (37)	0.48
Septic	2 (15)	7 (26)	
Hypovolemic	0	2 (7)	
Cardiogenic	1 (7)	1 (4)	
Invasive Ventilation, n (%)	7 (54)	14 (52)	0.98
PEEP, cm H_2_O	8 (5 − 9)	6 (5 − 10)	0.92
Lactate, mmol/L	2.6 (2.1 − 3.2)	2.6 (1.8 − 4.5)	0.75
Hemoglobin (mg/dL)	12.1 (9.6 − 13.3)	11.3 (9.8 − 13.1)	0.58
Enhanced OER,n (%)	4 (30)	6 (22)	0.71
Crystalloids,n (%)	6 (46)	12 (44)	0.73
Fluid volume (ml/kg)	8.27 (6.2 − 12.5)	8.62 (6.3 − 15.8)	0.76
Fluid rate (ml/min)	21.3 (16.9 − 32.5)	19.2 (16.1 − 23.5)	0.35
Baseline metabolic variables			
S_cv_O_2,_ %	63 (53 − 71)	70 (61 − 75)	0.18
P_va_CO_2,_ mmHg	4.6 (3.2 − 6.6)	8.7 (7.3 − 11.5)	< 0.01
P_va_CO_2_/C_av_O_2,_ mmHg/mL	1.0 (0.8 − 1.1)	2.1(1.7 − 2.4)	< 0.01
Oxygen delivery, mL/min/m^2^	355 (251 − 390)	442 (305 − 511)	0.07
Oxygen consumption, mL/min/m^2^	101 (89 − 119)	106 (92 − 160)	0.22
Oxygen extraction, %	33 (25 − 42)	28 (25 − 36)	0.34
Baseline hemodynamic variables			
Cardiac Index, L/min/m^2^	2.1 (1.7 − 2.4)	3.1 (1.9 − 3.6)	0.16
Stroke Volume, mL	44 (39 − 52)	56 (40 − 68)	0.22
Heart rate, beats/min	93 (75 − 105)	95 (79 − 112)	0.81
Pulse Pressure, mmHg	53 (39 − 68)	57 (45 − 65)	0.26
Mean Arterial Pressure, mmHg	77 (69 − 85)	80 (69 − 87)	0.64
Central Venous Pressure, mmHg	10 (3 − 11)	8 (4 − 11)	0.87

*PEEP* Positive end expiratory pressure, *S*_*cv*_*O*_*2*_ Central venous oxygen saturation, *P*_*va*_*CO*_*2*_ Veno-arterial carbon dioxide difference, *C*_*av*_*O*_*2*_ Arterio-venous oxygen difference ratio, *OER* Oxygen extraction ratio

**Table 2:** Hemodynamic and metabolic relative (d, %) and absolute changes (Δ) after fluid bolus (FB), baseline ratio of veno-arterial carbon dioxide difference to arterio-venous oxygen difference ratio (P_va_CO_2_/C_av_O_2_): low group has P_va_CO_2_/C_av_O_2_ < 1.4, and high group has P_va_CO_2_/C_av_O_2_ ≥ 1.4 mmHg/mL. Variables are expressed as the median (25–75% percentiles)

	Low Group	High Group	*p* values
**No of patients**	13	27	
d Hemoglobin (%)	-8.1 (-10.9 − -5.9)	-9.3 (-12.1 − -4.6)	0.97
Δ Hemoglobin (mg/dL)	-1.0 (-1.2 − -0.6)	-1.0 (-1.3 − -0.5)	0.94
**Metabolic variables changes**			
Δ S_cv_O_2_ (%)	-0.6 (-2.4 − 4.2)	1.8 (-3.6 − 3.8)	0.89
d Lactate (%)	-4.8 (-14.2 − 2.7)	9.2 (-3.1 − 16.6)	0.01
Δ Lactate (mmol/L)	-0.13 (-0.31 − 0.04)	0.26 (-0.11 − 0.52)	0.01
d P_va_CO_2_ (%)	43 (24 − 118)	-27 (-42 − -2)	< 0.01
Δ P_va_CO_2_ (mmHg)	2.6 (0.9 − 4.7)	-2.41 (-3.95 − -0.21)	< 0.01
d P_va_CO_2_/ C_av_O_2_ (%)	98 (41 − 139)	-15 (-34 − -2)	< 0.01
Δ P_va_CO_2_/ C_av_O_2_ (mmHg/mL)	0.92 (0.35 − 1.08)	-0.31 (-0.71 − -0.04)	< 0.01
d Oxygen delivery (%)	0.3 (-6.9 − 9.5)	4.5 (-7.3 − 14.5)	0.49
Δ Oxygen delivery (mL/min/m^2^)	1.39 (-19.6 − 27.3)	22.42 (-30.53 − 53.34)	0.58
d Oxygen consumption (%)	-1.6 (-5.6 − 1.9)	3.9 (-3.8 − 19.7)	0.11
Δ Oxygen consumption (mL/min/m^2^)	0.92 ( -4.32 − 3.14)	4.08 (-5.53 − 18.95)	0.34
Δ Oxygen extraction (%)	-0.54 (-3.61 − 3.33)	-1.1 (-3.13 − 2.12)	0.95
**Hemodynamic variables changes**			
d Cardiac Index (%)	8.8 (2.9 − 17.4)	13.4 (8.3 − 20.2)	0.34
Δ Cardiac Index (L/min/m^2^)	0.21 (0.06 − 0.43)	0.45 (0.21 − 0.54)	0.11
d Stroke Volume (%)	12.4 (0.47 − 15.5)	17 (5.5 − 22.4)	0.07
Δ Stroke Volume (mL)	5.16 (0.13 − 9.56)	10.23 (2.28 − 13.33)	0.14
d Heart rate (%)	-0.9 (-3.2 − 2.3)	-1.7 (-6.1 − 6.3)	0.73
Δ Heart rate (beats/min)	-1 (-3 − 2)	-2 (-6 − 3)	0.75
d Pulse Pressure (%)	6.3 (-1.4 − 15.1)	17.5 (-4.1 − 46.8)	0.32
Δ Pulse Pressure (mmHg)	2 (-1 − 5)	9 (-3 − 27)	0.24
d Mean Arterial Pressure (%)	1.7 (-7.7 − 13.6)	7.7 (-4.3 − 24.6)	0.21
Δ Mean Arterial Pressure (mmHg)	1 (-6 − 11)	6 (-4 − 19)	0.26
d Central Venous Pressure (%)	28 (12 − 143)	27 (10 − 62)	0.92
Δ Central Venous Pressure (mmHg)	2 (1 − 4)	2 (1 − 4)	0.96

*d* Delta, *S*_*cv*_*O*_*2*_ Central venous oxygen saturation, *P*_*va*_*CO*_*2*_ Veno-arterial carbon dioxide difference, *C*_*av*_*O*_*2*_ Arterio-venous oxygen difference ratio

### Association of P_va_CO_2_/C_av_O_2_ with significant blood-lactate decrease after FB

**Fig. 1:**
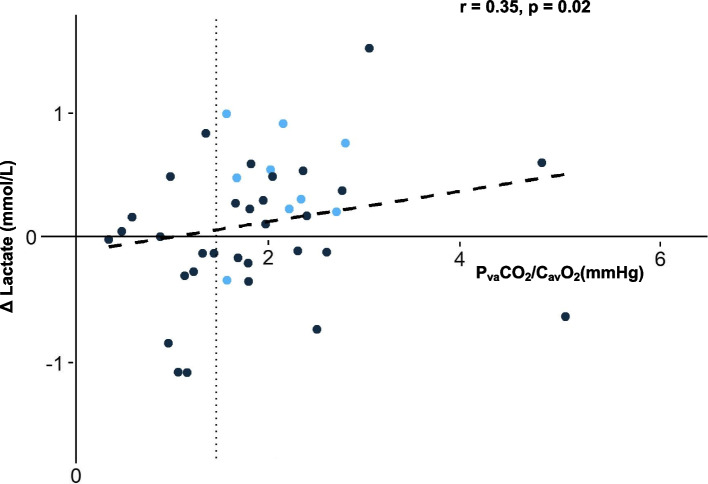
Changes in blood-lactate levels (Δ Lactate) during fluid bolus according to the baseline ratio of carbon dioxide veno-arterial difference to arterial-venous oxygen difference (P_va_CO_2_/C_av_O_2_). Blue points: patients with increase in oxygen consumption (VO_2_) ≥ 15%, black points: patients with change in oxygen consumption < 15%. Vertical dotted line corresponds to P_va_CO_2_/C_av_O_2_ of 1.4 mmHg/mL

There were 10 patients (25%) who had a significant decrease in blood-lactate levels after FB. All exept one of them had a P_va_CO_2_/C_av_O_2_ < 1 mmHg/mL (1.34 (1.01 − 1.71) mmHg/mL) (Figure S2). Logistic regression analyses did not demonstrate any association between the baseline P_va_CO_2_/C_av_O_2_ as a continuous value and significant decreases in lactate levels after FB (OR: 0.88 [95% CI: 0.39 − 1.98], *p* = 0.76). A P_va_CO_2_/C_av_O_2_ ≥ 1.4 was also not associated with significant decreases of blood lactate after FB (OR: 0.36 [95% CI: 0.08 − 1.59], *p* = 0.18). There was no evidence of moderation of the associations of P_va_CO_2_/C_av_O_2_ with decreases of blood lactate after FB by the changes of VO_2_ ≥ 15% (no interaction; *p* = 0.31). Preinfusion P_va_CO_2_/C_av_O_2_ was weakly and positively correlated with blood lactate changes during FB (*r* = 0.31, *p* = 0.04) (Fig. 1, Figure S2).

### Association of blood-lactate levels and P_va_CO_2_/C_av_O_2_ before and after FB

**Fig. 2:**
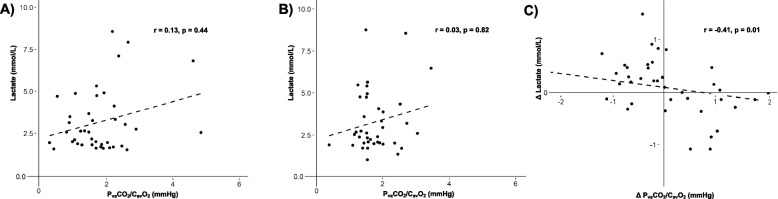
Correlation between blood-lactate levels and ratio of carbon dioxide veno-arterial difference to arterial-venous oxygen difference (P_va_CO_2_/C_av_O_2_) before (panel **A**) and after (panel **B**) fluid bolus and correlation of the changes (panel **C**) during FB

No significant correlation was observed between blood-lactate levels before and after FB. A statistically significant but weak negative correlation was observed between the changes in blood lactate and P_va_CO_2_/C_av_O_2_ (*r* = -0.41, *p* = 0.01) (Fig. 2, Figures S3 & S4).

### Association of oxygen consumption and blood lactate changes during FB

**Fig. 3:**
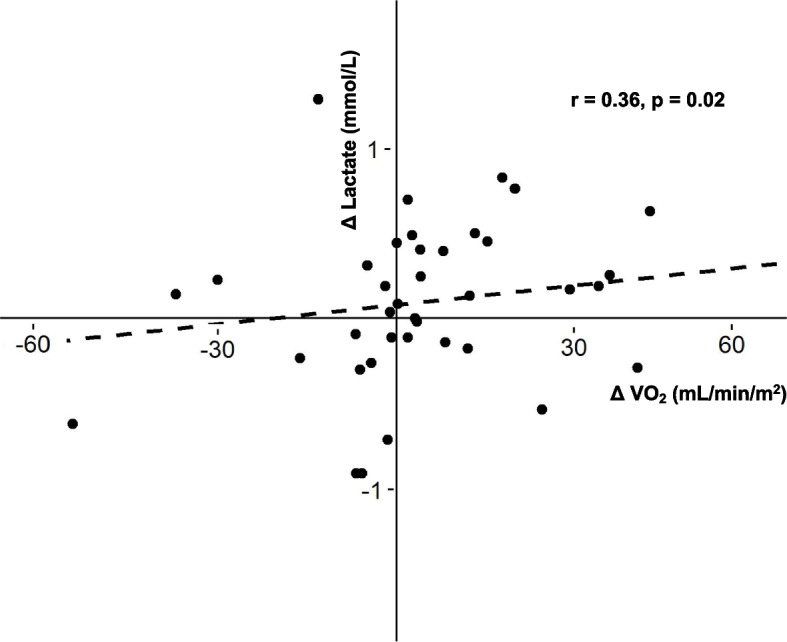
Correlation between changes in blood-lactate levels (Δ Lactate) and changes in oxygen consumption (Δ VO_2_) during fluid bolus

There were 10 patients (25%) who had a significant increase in VO_2_ after FB, and none of them had low P_va_CO_2_/C_av_O_2_ before FB. Among patients with elevated baseline P_va_CO_2_/C_av_O_2_, no statistically significant differences were observed in baseline values of P_va_CO_2_ (8.7 (7.7 − 11.2) mmHg vs. 8.9 (7.2 − 10.1) mmHg, *p* = 0.96) or in baseline values of S_cv_O_2_ (70 (61 − 78) % vs. 69 (61 − 74) %, *p* = 0.76) between those with or without an increase in VO_2_. Changes in VO_2_ were weakly and positively correlated with lactate changes (*r* = 0.36, *p* = 0.02) (Fig. 3, Figure S5).

## Discussion

The results of this study can be summarized as follows: 1) in critically ill patients with hyperlactatemia, elevated P_va_CO_2_/C_av_O_2_ is not associated with decreases in blood-lactate levels after FB and cannot be used to predict them, 2) increases in oxygen consumption observed only in patients with elevated baseline P_va_CO_2_/C_av_O_2_ were not associated with blood lactate decreases after FB and 3) in patients with hyperlactatemia, blood-lactate levels and changes during FB were not corelated with P_va_CO_2_/C_av_O_2_ values and changes.

Previous studies have suggested using the P_va_CO_2_/C_av_O_2_ ratio measured after the end of resuscitation for predicting failure for decreasing blood-lactate levels in patients with hyperlactatemia [15–17]. Similar to these studies, we found that decreasing blood-lactate levels are less likely in patients with elevated P_va_CO_2_/C_av_O_2_ before FB. Conversely, and similar to another study [15], patients with low baseline P_va_CO_2_/C_av_O_2_ had a higher likelihood for decreasing blood lactate levels. Thus, the results of our study extend the knowledge about the FB effects on blood lactate in critically ill patients according to baseline P_va_CO_2_/C_av_O_2,_ suggesting that FB has limited effects on decreasing lactate levels in patients with elevated baseline P_va_CO_2_/C_av_O_2_. Additionally, the reduction in blood lactate levels observed in patients with normal baseline P_va_CO_2_/C_av_O_2_ values may not be associated with an improvement of aerobic metabolism after FB.

Herein, P_va_CO_2_/C_av_O_2_ was used as an alternative of respiratory quotient. The failure of high P_va_CO_2_/C_av_O_2_ to predict decreases in blood lactate levels after FB could be due to the unreliable correlation of this ratio with the occurrence of anaerobic metabolism in this mixed population of critically ill patients [18]. Similar to previous studies [8, 9] several patients with hyperlactatemia and high P_va_CO_2_/C_av_O_2_ increased VO_2_ after FB, which implies oxygen delivery/consumption dependence. However, high oxygen extraction state was observed only in few patients with elevated P_va_CO_2_/C_av_O_2_, which may contradict this hypothesis.Therefore, we conclude that in our cohort of non-selected critically ill patients with hyperlactatemia, elevated P_va_CO_2_/C_av_O_2_ may reflect not only anaerobic metabolism as a result of tissue hypoxia (i.e. hypoperfusion), but also as a result of tissue dysoxia (i.e. impairment in oxygen utilization) [19].

Significant decreases in blood lactate after FB were observed in patients with P_va_CO_2_/C_av_O_2_ < 1.4 mmHg/mL, but not in the patients with P_va_CO_2_/C_av_O_2_ < 1 mmHg/mL. P_va_CO_2/_C_av_O_2_ ≥ 1.4 mmHg/mL was defined abnormal based on the previous studies that have demonstrated that this cut-off can predict persistent hyperlactatemia in critically ill patients [6, 15]. Incidentally, other authors have suggested higher cut-off values to predict increases in VO_2_ after FB [8, 9, 20]. However, in another study, a cut-off of > 1 mmHg/mL was found to adequately predict mortality [17]_._ Hence, our results suggest that the anaerobic threshold of critically ill patients with hyperlactatemia may vary across the patients [21]. Nevertheless, a value of P_va_CO_2_/C_av_O_2_ < 1 mmHg/mL may be used to exclude anaerobic metabolism and decreasing blood lactate levels after FB.

Increases in oxygen consumption after FB were not associated with a decrease in blood-lactate levels. Notably, we found that the patients who had an increase oxygen consumption after FB were less likely to present a significant decrease in blood-lactate levels. Different factors can explain this phenomenon. Calculation of VO_2_ based on the reverse Fick principle may not be accurate [22, 23]. Inadequate hemodynamic resuscitation can be an additional explanation, even though a sufficient dose of fluid at high rate was administrated [24]. Furthermore, increased tissue perfusion during FB may cause a paradoxical elevation in blood lactate levels due to ‘washout’ phenomenon [25–28] or accelerated aerobic glycolysis [29, 30]. Of note, a weak correlation between VO_2_ and blood lactate changes was observed. Thus, based on our results, high P_va_CO_2_/C_av_O_2_ before FB is associated with VO_2_ dependency on DO_2_, similar to previous studies [8, 9], but also with failure of FB to decrease blood lactate. In these patients, whether no change or even increase in blood lactate levels indicates FB failure to improve peripheral perfusion should be further evaluated in future studies.

Not surprisingly, the majority of the patients with hyperlactatemia had an elevated P_va_CO_2_/C_av_O_2_ since either of these variables increases due to anaerobic metabolism. Nevertheless, there was no correlation between P_va_CO_2_/C_av_O_2_ and blood-lactate levels. Hence, our results suggest that P_va_CO_2_/C_av_O_2_ can be used as a complementary marker for the evaluation of patients with hyperlactatemia and the effects of FB. For instance, we observed a negative correlation of changes in blood lactate and P_va_CO_2_/C_av_O_2_ during FB. A plausible explanation for this phenomenon could be that decreases in blood lactate illustrate an improvement in tissue oxygenation, and P_va_CO_2_/C_av_O_2_ illustrates oxygen debt repayment after perfusion improvement [31, 32].

The strength of this study was that we evaluated the predictive value of P_va_CO_2_/C_av_O_2_ in a non-selected critically ill population with mild hyperlactatemia treated with FB. Patients in this cohort presented a high range of P_va_CO_2_/C_av_O_2_, and a significant number of patients had low P_va_CO_2_/C_av_O_2_. We evaluated changes in blood lactate close to the time of the FB in stable conditions, and non-major variation in metabolism was expected.

Nevertheless, this study has several limitations. First, no formal sample power calculation was done and not predictive test were performed. However, the results are in the opposite direction of our hypothesis, and the possibility of finding different results with a higher sample size is low. Additionaly, based on our findings we conclude that P_va_CO_2/_C_av_O_2_ cannot have clinical relevant predictive value for decreasing blood lactate levels after FB. Second, therapeutic interventions that can affect lactate levels before and after FB were not standardized. However, all the patients were treated under standard local therapeutic strategies. Third, only central venous and not mixed venous-to-arterial carbon dioxide tension differences were evaluated. Fourth, other parameters that can affect the amount P_va_CO_2_ such as temperature, metabolic acidosis, and Haldane effect were not assessed in this study. Fifth, we did not evaluate that within-subject variability might significantly influence our results, as relatively low values of baseline blood lactate were observed. Sixth, possible liver dysfunction effects on lactate metabolism was not evaluated.

## Conclusions

Elevated P_va_CO_2_/C_av_O_2_ are not associated with FB induced blood lactate decreases. In this small cohort of critically ill patients with hyperlactatemia, high preinfusion P_va_CO_2/_C_av_O_2_ was associated with less decrease in blood-lactate levels after FB.

## Supplementary Information

**Additional file 1:** **Figure S1. **Flow chart of patients selection. **Table S1. **Principal reasons for fluid bolus. **Figure S2.** Prevalence of the patients who had significant decrease in blood lactate levels during fluid bolus (FB) according to pre-infusion arterial-venous oxygen difference ratio (P_va_CO_2_/C_av_O_2_). Dotted line: trendline (regression analysis). **Figure S3.  **Changes in blood lactate levels ( Δ Lactate) during fluid bolus according to the baseline carbon dioxide veno-arterial difference to arterial-venous oxygen difference ratio (P_va_CO_2_/C_av_O_2_), in patient without (Pannel A) or with (Pannel B) enhanced oxygen extraction. **Figure S4. **Correlation between blood lactate levels and arterio-venous oxygen difference ratio(P_va_CO_2_/ C_av_O_2_) before (panel A) and after (panel B) fluid bolus (FB) and correlation of the changes (panel C) during FB in patients without enhanced oxygen extraction. **Figure S5. **Correlation between blood lactate levels and arterio-venous oxygen differenceratio (P_va_CO_2_/ C_av_O_2_) before (panel A) and after (panel B) fluid bolus (FB) and correlation of the changes (panel C) during FB in patients with enhanced oxygenextraction. **Figure S6.**  Correlation between blood lactate levels changes and oxygen consumption changes during fluid bolus, in patient without (Pannel A) or with (Pannel B) enhanced oxygen extraction. 

## Data Availability

All data generated or analysed during this study are included in this published article and its supplementary information files.

## Abbreviations

P_va_CO_2_: Carbon dioxide veno-arterial difference
C_av_O_2_: Oxygen arterial-venous difference
VO_2_: Oxygen consumption
FB: Fluid bolus
APACHE: Acute Physiology and Chronic Health Evaluation
DO_2_: Oxygen delivery
OER: Oxygen extraction ratio
