# Supplementary material for: Ratio of carbon dioxide veno-arterial difference to oxygen arterial-venous difference is not associated with lactate decrease after fluid bolus in critically ill patients with hyperlactatemia: results from a prospective observational study
Source: BMC Anesthesiol. 2023 Jan 31;23:37. doi: 10.1186/s12871-023-01993-6 (PMC9887917; doi:10.1186/s12871-023-01993-6)

**Ratio of carbon dioxide veno-arterial difference to oxygen arterial-venous difference is not associated with lactate decrease after fluid bolus in critically ill patients with hyperlactatemia: results from a prospective observational study**

[*Keitiane KAEFER,MD*](https://pubmed.ncbi.nlm.nih.gov/?sort=date&term=Kaefer+K&cauthor_id=35561559)*, Charalampos PIERRAKOS, MD,*   *Thomas NGUYEN, MD, Dimitrios VELISSARIS, MD, Rachid ATTOU, Jacques DEVRIENDT, MD*

*Sabino SCOLLETTA MD, PhD, Fabio Silvio TACCONE,* *MD, PhD*

Supplement

**Figure S1.** Flowchart of patients selection


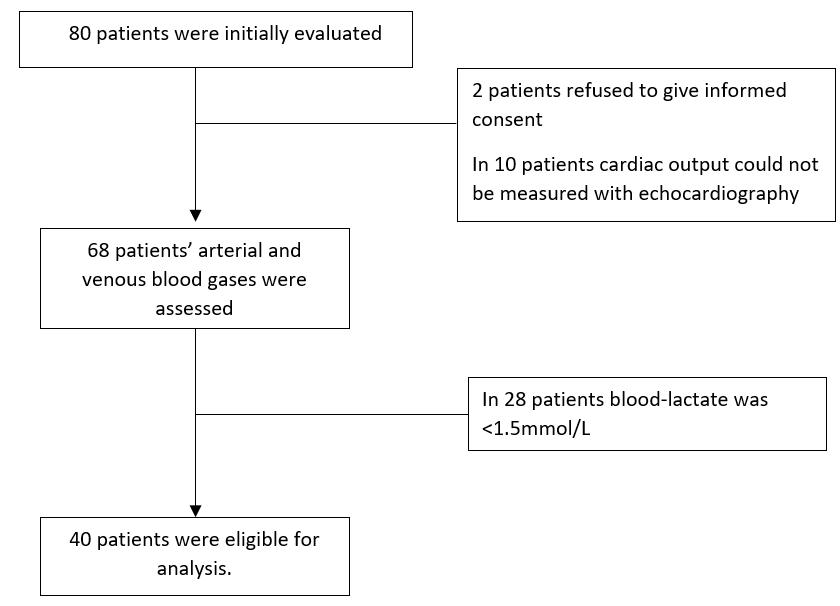


**Table S1.** Principal reasons for fluid bolus

| **Reason for fluid bolus** | **No of the patients** |
| --- | --- |
| Hypotension (%) | 8 (20) |
| Weaning of noradrenaline (%) | 5 (13) |
| Oliguria (%) | 10 (25) |
| Persistent elevated levels of blood lactate | 17 (42) |

**Figure S2.** Prevalence of the patients who had significant decrease in blood lactate levels during fluid bolus (FB) according to pre-infusion arterial-venous oxygen difference ratio (P_va_CO_2_/C_av_O_2_). Dotted line: trendline (regression analysis)


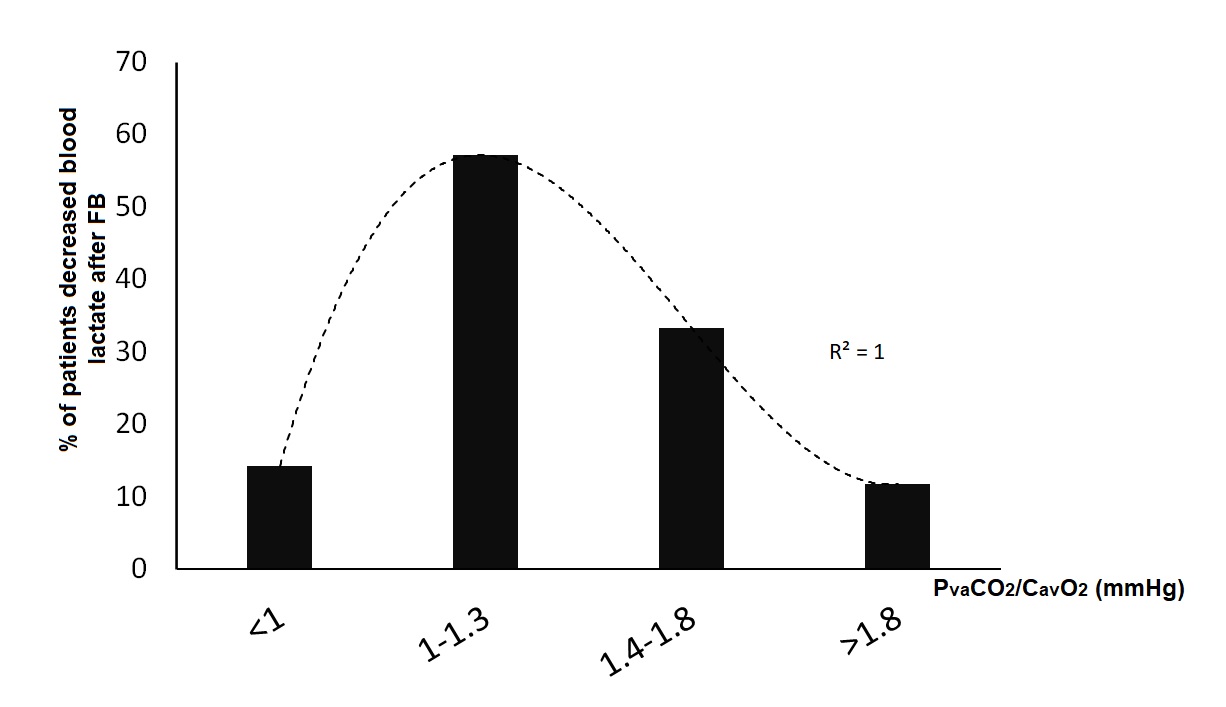


**Figure S3.** Changes in blood lactate levels ( Δ Lactate) during fluid bolus according to the baseline carbon dioxide veno-arterial difference to arterial-venous oxygen difference ratio (P_va_CO_2_/C_av_O_2_), in patient without (Pannel A) or with (Pannel B) enhanced oxygen extraction.


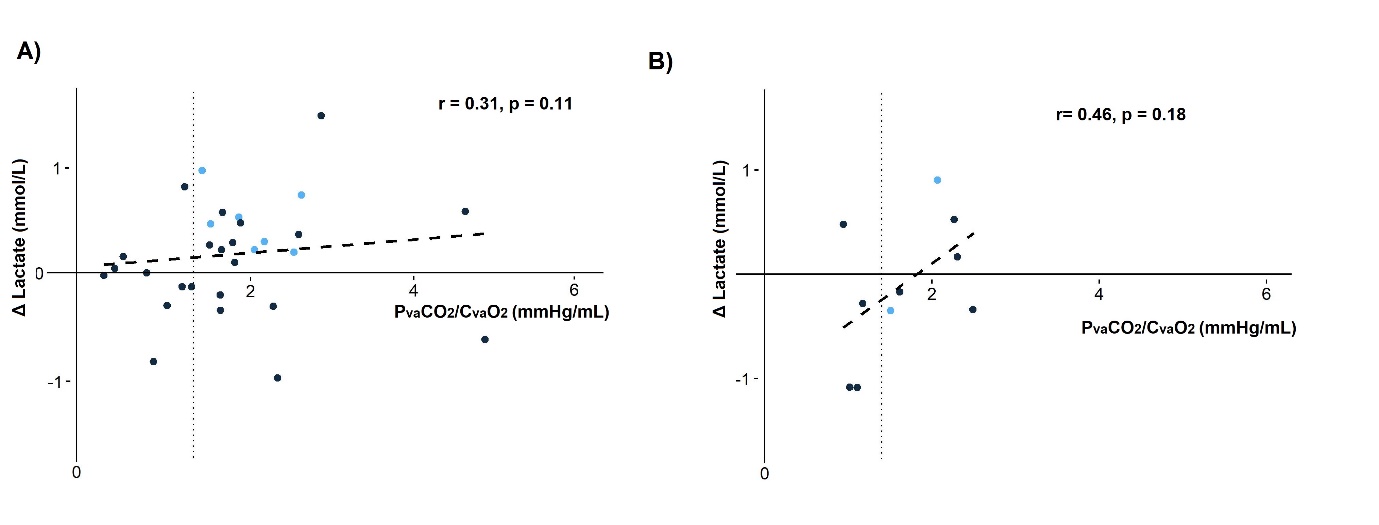


**Figure S4.** Correlation between blood lactate levels and arterio-venous oxygen difference ratio (PvaCO2/ CavO2) before (panel A) and after (panel B) fluid bolus (FB) and correlation of the changes (panel C) during FB in patients without enhanced oxygen extraction.


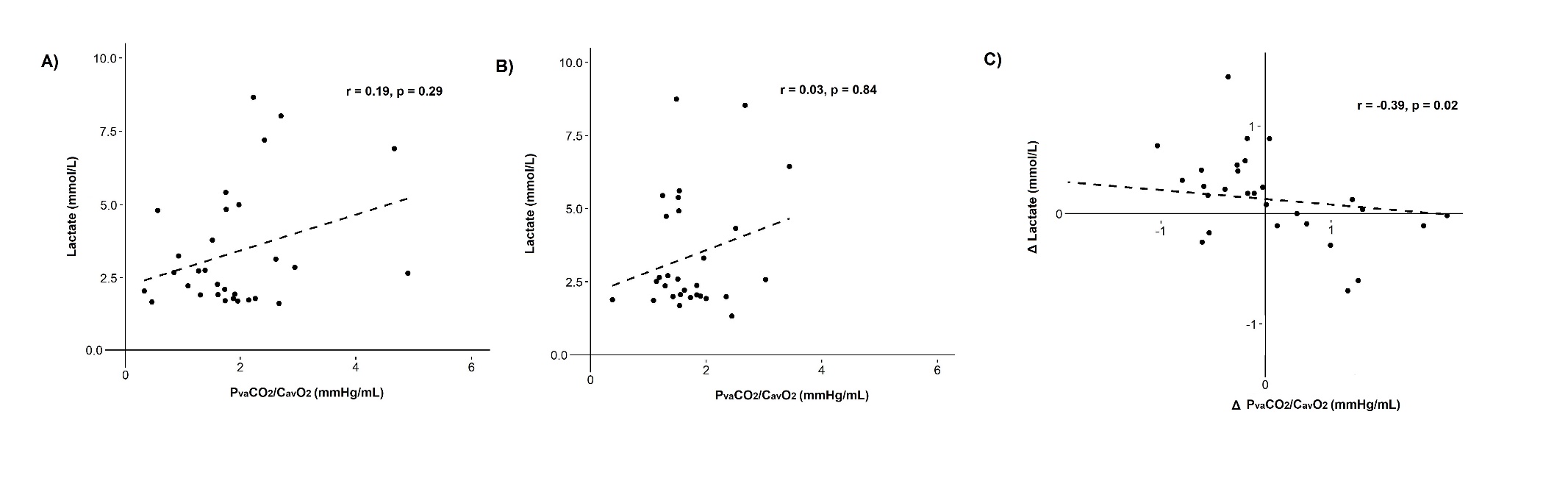


**Figure S5.** Correlation between blood lactate levels and arterio-venous oxygen difference ratio (PvaCO2/ CavO2) before (panel A) and after (panel B) fluid bolus (FB) and correlation of the changes (panel C) during FB in patients with enhanced oxygen extraction.


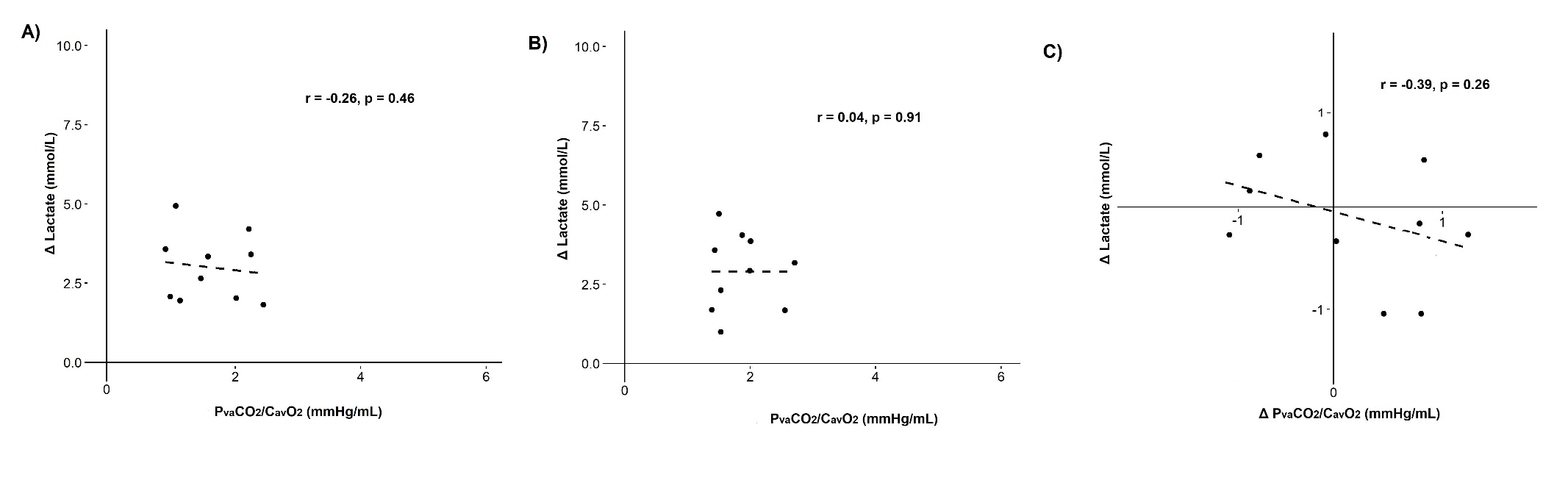


**Figure S6.** Correlation between blood lactate levels changes and oxygen consumption changes during fluid bolus , in patient without (Pannel A) or with (Pannel B) enhanced oxygen extraction.


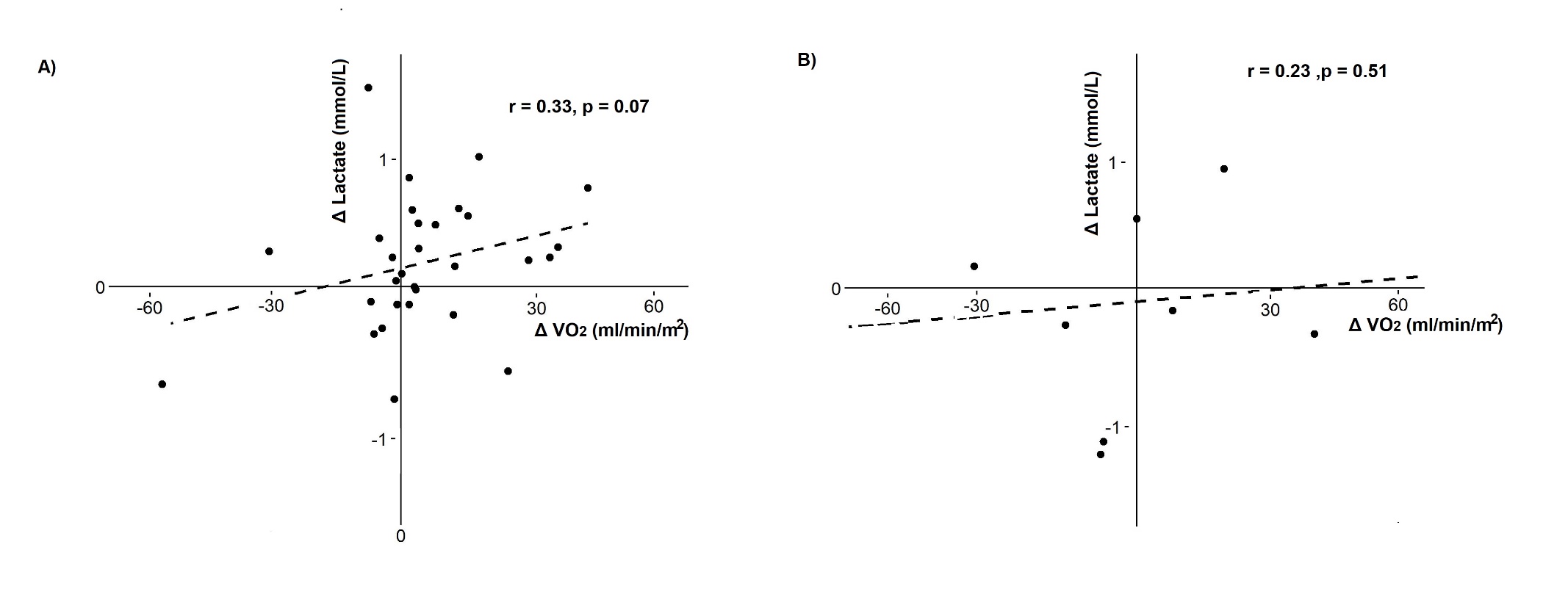

Supplement: Supplementary file 1 — Additional file 1: Figure S1. Flow chart of patients selection. Table S1. Principal reasons for fluid bolus. Figure S2. Prevalence of the patients who had significant decrease in blood lactate levels during fluid bolus (FB) according to pre-infusion arterial-venous oxygen difference ratio (PvaCO2/CavO2). Dotted line: trendline (regression analysis). Figure S3. Changes in blood lactate levels ( Δ Lactate) during fluid bolus according to the baseline carbon dioxide veno-arterial difference to arterial-venous oxygen difference ratio (PvaCO2/CavO2), in patient without (Pannel A) or with (Pannel B) enhanced oxygen extraction. Figure S4. Correlation between blood lactate levels and arterio-venous oxygen difference ratio(PvaCO2/ CavO2) before (panel A) and after (panel B) fluid bolus (FB) and correlation of the changes (panel C) during FB in patients without enhanced oxygen extraction. Figure S5. Correlation between blood lactate levels and arterio-venous oxygen differenceratio (PvaCO2/ CavO2) before (panel A) and after (panel B) fluid bolus (FB) and correlation of the changes (panel C) during FB in patients with enhanced oxygenextraction. Figure S6. Correlation between blood lactate levels changes and oxygen consumption changes during fluid bolus, in patient without (Pannel A) or with (Pannel B) enhanced oxygen extraction. [file 12871_2023_1993_MOESM1_ESM.docx]
